# Gender-related differences in the effects of *Inonotus obliquus* polysaccharide on intestinal microorganisms in SD rats model

**DOI:** 10.3389/fvets.2022.957053

**Published:** 2022-09-20

**Authors:** Binhong Hu, Xinyue He, Jin Tan, Yichuan Ma, Gang Wang, Songqing Liu, Mingyue Li, Yanping Guo, Rong Sun, Mengxue Sun, Xin Deng, WenJing Zhou, Xue Lv

**Affiliations:** ^1^College of Chemistry and Life Sciences, Chengdu Normal University, Chengdu, China; ^2^Sichuan Provincial Key Laboratory for Development and Utilization of Characteristic Horticultural Biological Resources, Chengdu Normal University, Chengdu, China

**Keywords:** *Inonotus obliquus*, polysaccharide, intestinal microflora, 16S rRNA, SD rats

## Abstract

Natural edible fungal polysaccharides are of research and application value for the prevention of diseases by improving the microenvironment within the intestine. *Inonotus obliquus* polysaccharide (IOP) extracts have strong antioxidant, anti-inflammatory, and other biological activities, and as such, it could be used as prebiotics to improve the viability of intestinal microbes, maintain intestinal homeostasis and improve intestinal immunity. The effects of sex on intestinal microbiota after IOP absorption was determined. In this study, IOP had different effects on the intestinal flora of male and female rats, with the diversity and richness showing opposite changes. At the same time, after IOP intervention, changes in the dominant intestinal flora of female rats was less compared with that of males. In addition, while *Clostridia, Lactobacillus* and *Roseburia* were the dominant intestinal microbes in female rats, males had mainly *Bacteroidota* from different families and genera, along with an increasing proportion of *Muribaculaceae* from different families and genera. IOP could further regulate the intestinal microenvironment of male and female SD rats by enhancing the vitality of their dominant microorganisms, and for both sexes, this enabled the screening of dominant microflora that were conducive to the balance of the intestinal flora. These results help to understand the effects of sex-related differences on the composition of the intestinal microbiota as well as on diseases.

## Introduction

Gut microbes are considered to be a dynamic organ that plays a vital role in the digestion of food components as well as in host immune responses (1). Clear individual differences in the composition of human gut microbes have been identified by genome-based assessments (2), and simultaneously, conclusive evidence have shown that diversification of the gut microbiome can lead to diseases such as asthma (3) and cancer (4). Nevertheless, correlational research of intestinal microbes between individuals still has knowledge gaps. It is particularly important to understand how the composition of the gut microbiota vary between individuals as this would have significant implications for the prevention of sex-related diseases.

Sex hormones are associated with the composition of human gut microbes (5). Sex-related differences in intestinal microbiome composition in animals have been reported and the studies indicated that sex-related differences in gut microbes do not appear until after puberty (6, 7). The gut microbiota is sex-dependent and may be directly affected by sex hormones. In addition, these hormones interact with environmental factors such as dietary habits and antibiotic treatment to influence the microbiome (8). The body's dynamic changes in response to estrogen or other hormones, is also potentially linked to disease. In this context, some studies have shown that the microbiome is related to sex dimorphism and sex-specific rhythm of the liver, and this could be caused by the secretion of growth hormone (GH) and the regulation of sexual maturity (9). The findings of these studies provide a basis that explains sex-related differences in susceptibility to certain diseases. The occurrence and development of many diseases are linked to immunity and metabolism. Given that the intestinal flora plays an important role in maintaining an internal environmental balance that regulates immunity and metabolism and defends against pathogens (10, 11), it is likely that prebiotics may help to understand the impact of the gut microbiome on disease (12).

*Inonotus obliquus* is a natural edible and medicinal fungus which grows mainly in the colder regions of Asia and Europe. Its metabolites have been shown to possess a wide range of biological properties such as anti-inflammatory, antioxidant and antiviral (13–15), and as such, they have the potential to be used for preventing cancer, diabetes, cerebrovascular or other diseases (13, 16, 17). However, of these metabolites, most researchers have been paying greater attention to the biological effects of *Inonotus obliquus* polysaccharides on the body where they form a biological barrier that protects hosts from pathogens (18, 19). The composition of polysaccharides as well as their effects on gut health vary greatly, and consequently, differences in their effects on gut microbiome between individuals need to be considered.

To determine how the effects of *Inonotus obliquus* polysaccharide consumption vary between sexes, the composition of intestinal microbiota in SD rats was assessed, after they had been fed a normal diet or a rich *Inonotus obliquus* polysaccharide one. The relationship between sex and the composition of intestinal microbiota as well as the distribution of specific taxa was studied after consuming the polysaccharides. It is expected that the results of this study will help to better understand the effects of polysaccharides on gut microbiota in order to reduce risk of diseases and mitigate specific health risks.

## Materials and methods

### Animals and reagents

All laboratory procedures, including those related to animal handling, welfare and euthanasia, were carried out in accordance with the guidelines and regulations of Animal Research Reporting of *In Vivo* Experiments (ARRIVE) and approved by the Animal Care Office of Chengdu Normal University, Chengdu, China (No: CDNU-2021092614M). SD rats, free from pathogens, were purchased from the Chengdu Dossy Experimental Animals Co., Ltd while *Inonotus obliquus* was obtained from Suifenhe Market in the Heilongjiang Province.

### The extraction of *Inonotus obliquus*

Traditional extraction methods based on hot water extraction, followed by centrifugation, were applied (20, 21). For this purpose, *Inonotus obliquus* (IO) was first crushed before being degreased with petroleum ether. The polysaccharide residue was then dried at low temperature, heated and boiled with distilled water and subsequently filtered. After repeating the above process three times, all resulting filtrates were mixed, the solvent was recovered under pressure, and 1% of trichloroacetic acid was added to precipitate proteins. The mixture was then centrifuged and concentrated into a liquid extract before overnight precipitation at 0°C with anhydrous ethanol. The alcohol-containing solution was centrifuged at high speed to obtain a crude extract which was subsequently washed 2–3 times with a small amount of anhydrous ethanol to obtain crude polysaccharide. DEAE- cellulose anionic column chromatography was used to specifically isolate acidic and neutral mucopolysaccharides. After removing the pigment, 2% sodium nitrite solution was added in a ratio of 1:2 before heating the mixture in a water bath at 80°C. This was followed by the addition of activated carbon and after stirring, the solution was heated again for 1 h before being left overnight. It was eventually centrifuged and freeze-dried to obtain the decolorized polysaccharides.

### Monosaccharide and uronic acid compositional analysis

The monosaccharide and uronic acid composition of IOP was analyzed by HPLC (Agilent 1200, USA). For this purpose, 3.41 mg of IOP was carefully taken into an ampoule before adding 0.5 ml of 4 M trifluoroacetic acid. Nitrogen was then pumped into the ampoule for 5 min before sealing it at 121°C for 2 h. The trifluoroacetic acid was blown dry with nitrogen, and 0.5 ml of 0.5 M PMP as well as 0.5 ml of 0.3 M NaOH were added. The mixture was then kept in a water bath at 70°C for 1 h, and after allowing it to cool, 0.5 ml of 0.3 M HCl was added. This was followed by chloroform extraction three times, with the supernatant subsequently centrifuged after the third extraction. The final sample was passed through a 0.22 μm membrane before eventually injecting 20 μl into the injection bottle for analysis at 245 nm. Standard monosaccharides and uronic acids were also analyzed, and their standard curves are shown in Table 1.

**Table 1:** Regression equations and *R*^2^ of monosaccharides and uronic acids.

**Monosaccharides or** **uronic acids**	**Regression equation**	***R*** **^2^**
Man	*y* = 0.0002*x –* 0.0069	1
Rham	*y* = 0.0001*x –* 0.06978	0.99995
Ribose	*y* = 0.00006*x –* 0.01788	0.99983
Glu A	*y* = 0.0001*x* + 0.0027	1
Gal A	*y* = 0.0001*x –* 0.0124	0.9999
Gal	*y* = 0.00033*x –* 0.01613	0.99929
Glu	*y* = 0.0001*x –* 0.0113	1
Xyl	*y* = 0.0005*x –* 0.0073	0.9997
Ara	*y* = 0.0008*x –* 0.0095	0.9999
Fuc	*y* = 0.0003*x –* 0.0032	1

### Experimental treatment

**Figure 1:**
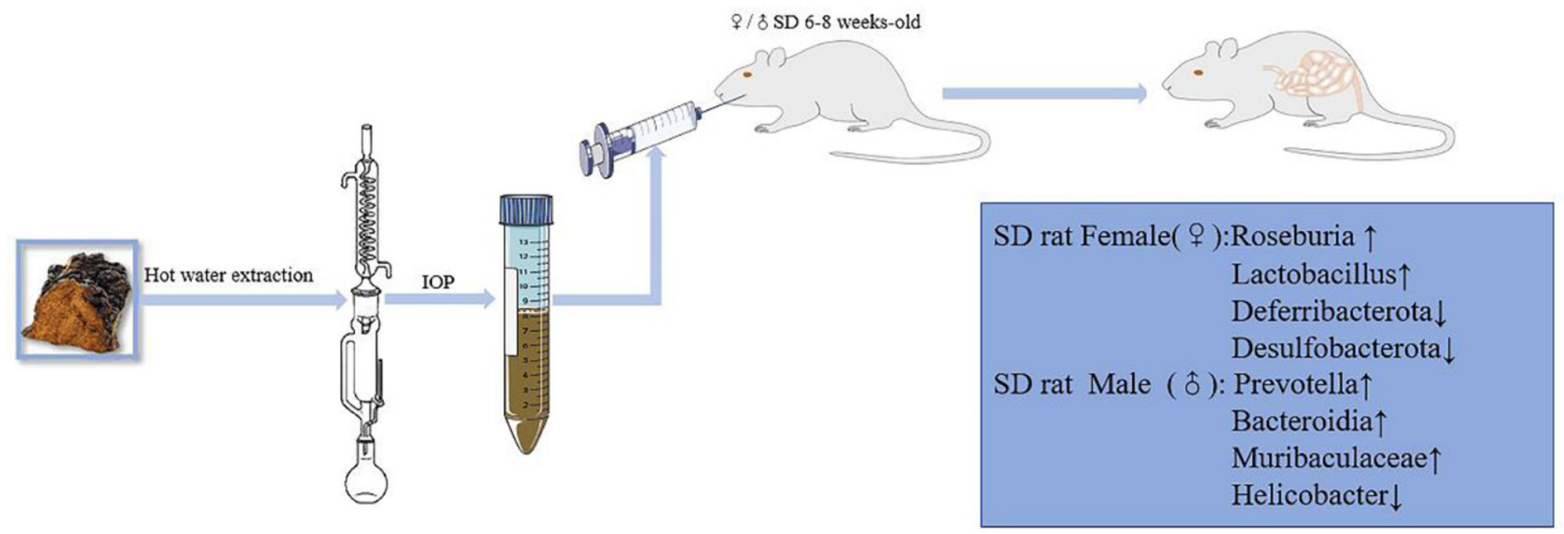
Animal experiment process. IOP increased the diversity and richness of intestinal microbes in male and female SD rats, while also affecting the abundance of specific microbes differently in each sex.

Forty-eight SD rats (female: 150 ± 10 g, male: 180 ± 10 g) were placed in an environment with a 12-h light/dark cycle, a temperature of 25 ± 3°C and a relative humidity of 75 ± 5%. The animals were also provided adequate food and water. Rats of each sex were then randomly divided into a control group and an IOP group after 7 days of adaptation. The IOP group was given IOP (60 mg/kg) by timing intragastric administration, while the control group was simultaneously given an equal proportion of Stroke-Physiological Saline Solution. The rats were eventually stunned with ether before being killed by neck dislocation after 4 weeks of treatment. The colorectal content was then collected under sterile conditions and stored at −80°C after rapid-freezing with liquid nitrogen (Figure 1).

### DNA extraction and amplification

Bacterial DNA was first isolated from colorectal content by using the MagPure Soil DNA LQ kit (Guangdong Magen, China) as per the manufacturer's instructions. DNA concentration and integrity were then determined with a NanoDrop 2000 spectrophotometer (Thermo Fisher Scientific, Waltham, MA, USA) and agarose gel electrophoresis. Using TksGflflex DNA polymerase (Takara, R060B) as well as the universal primer pairs 343f (5′-TACGGRAGGCAGCAG-3′) and 798r (5′-AGGGTATCTAATCT-3′), PCR amplification of the V3–V4 hypervariable region of bacterial 16S rRNA gene was subsequently performed in 25 μl reaction mixtures. The reverse primers contained sample barcodes and both were also connected to Illumina sequencing adapters.

### Library construction and sequencing

PCR products were purified with Agencourt AMPure XP beads (Beckman Coulter Co., USA) and quantified with Qubit dsDNA detection kits. Sequencing was then performed on an Illumina NovaSeq6000 platform to generate 250 bp paired-end reads (Illumina Inc., San Diego, California; Shanghai OE Biotech Co., Shanghai, China).

### Bioinformatics analysis

Paired-end reads were preprocessed using Trimmomatic software (22) to detect and cut off ambiguous bases (N). The sliding window pruning method was used to cut off low-quality sequences with an average mass score below 20. After trimming, FLASH software was used to assemble paired-end reads (23) according to the following parameters: a minimum overlap of 10 bp, a maximum overlap of 200 bp and a maximum mismatch rate of 20%. Further denoising of the sequences involved discarding readings with ambiguous or homologous sequences as well as those below 200 bp. QIIME software (version 1.8.0) (24) was then used to retain 75% of bases above Q20, while VSEARCH was applied to detect and remove both chimeras and primer sequences. Clean reads were clustered at a 97% similarity cutoff value to generate operational classification units (OTUs) (25), with the QIIME package subsequently used to select representative reads for each OTU. All representative reads were annotated against the Silva database (version 132) using the RDP classifier (confidence threshold 70%) (26). Alpha-diversity indices, including the Chao1 index (27) and the Shannon index (28) were then used to estimate microbial diversity in samples of colon content before comparing the results based on Wilcoxon rank sum test. The Unifrac distance matrix, as estimated by QIIME software, was eventually used for unweighted Unifrac principal coordinate analysis (PCoA) as well as for constructing phylogenetic trees.

## Results

### Monosaccharide and uronic acid composition of IOP

**Figure 2:**
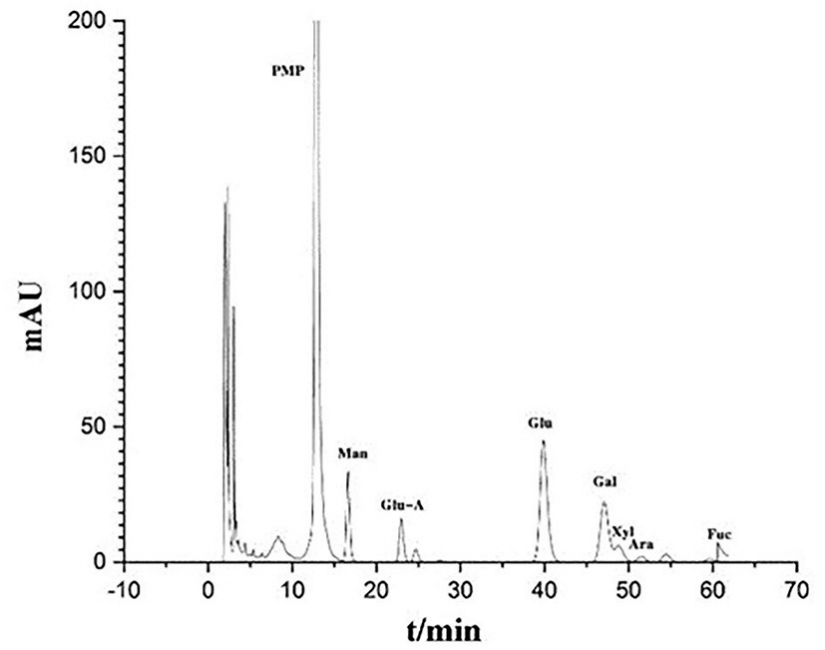
Monosaccharide and uronic acid composition of IOP as determined by HPLC.

In order to further understand the structure-activity relationship of polysaccharides from Inonotus obliquus, the compound was purified. During the purification process, protein, pigments and acidic sugars were removed to obtain neutral sugars. Finally, the chromatogram of the polysaccharides was obtained by HPLC (Figure 2). It was observed that the purified polysaccharides were composed of Man, Glu A, Glu, Gal, Xyl, Ara and Fuc.

### The effects of IOP on the overall abundance of intestinal microbes

**Figure 3:**
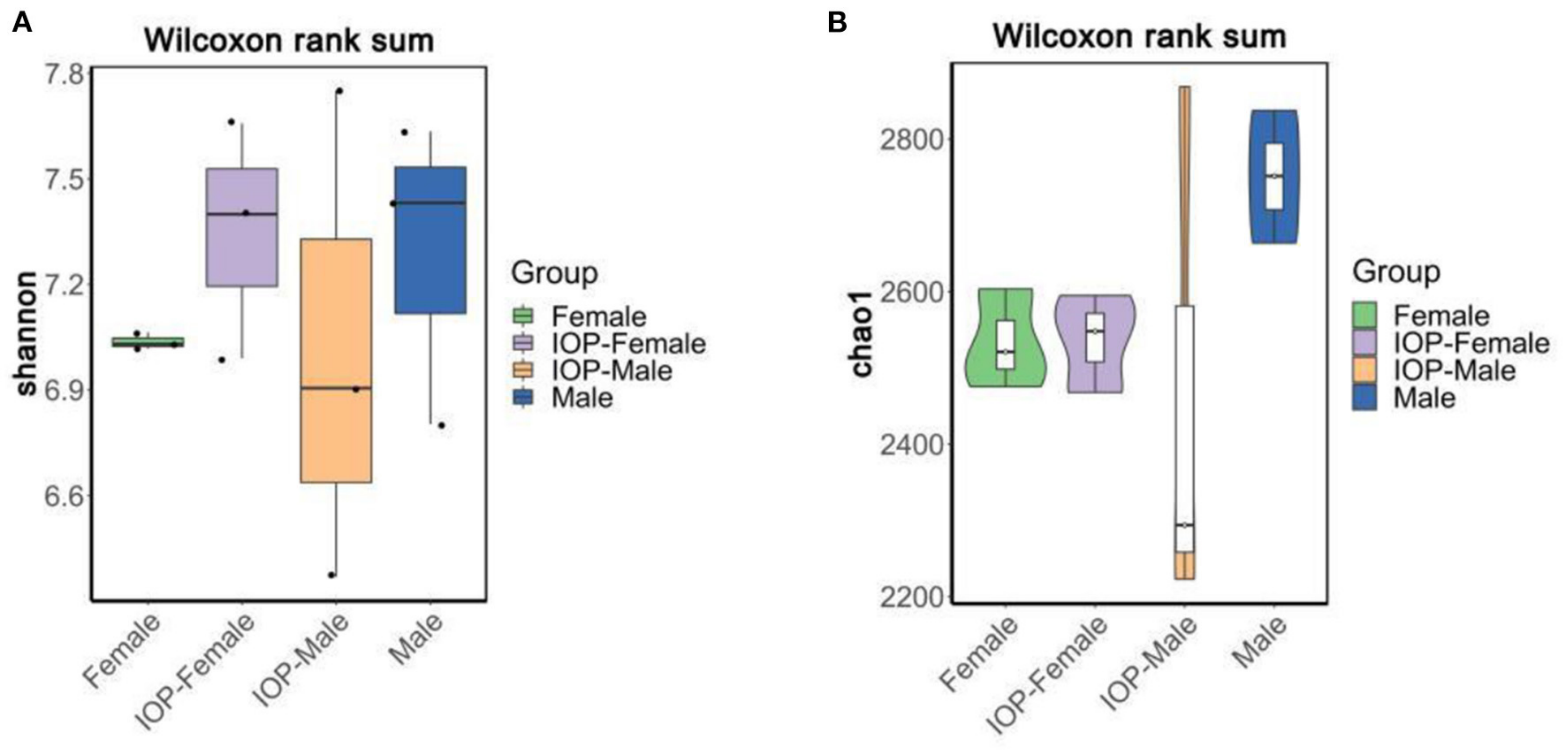
Alpha-diversity compared based on Wilcoxon rank sum test. **(A)** Shannon boxplots of all SD rats. **(B)** Chao1 violin plots of all SD rats.

**Figure 4:**
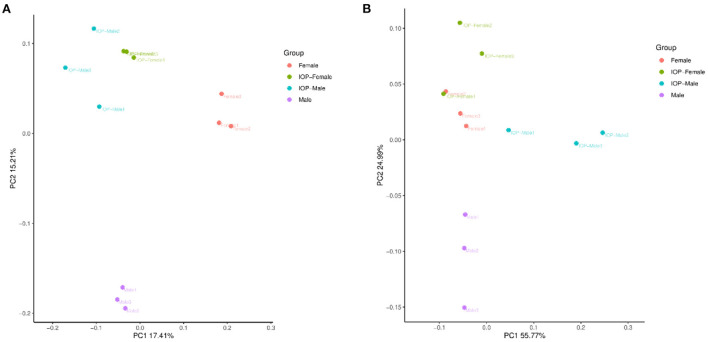
Beta diversity of samples based on Adonis test. **(A)** PCoA plots based on unweighted unifrac distances for all SD rats. **(B)** PCoA plots based on weighted unifrac distances for all SD rats.

Between 71,485 and 75,214 clean tags were obtained after quality control and the removal of chimeras from the original sequences. After Operational taxonomic units (OTU) classification, the number of OTUs in 48 samples ranged from 1,673 to 2,342. Results for the Shannon index and Chao1 index indicated that the diversity and richness of the IOP group were significantly changed compared with the control group (Figure 3). Beta-diversity, combined with adonis analysis (unweighted *R* = 0.45, *P* = 0.001; weighted *R* = 0.75, *P* = 0.001), further showed that the differences between groups were greater than for within groups, with significant differences being noted between the IOP group and the control group. Based on the unweighted (qualitative) and weighted (quantitative) UniFrac distance measures in PCoA analysis, significant differences in weighted and unweighted UniFrac distances between male and female rats of the control group were also found (Figure 4).

**Figure 5:**
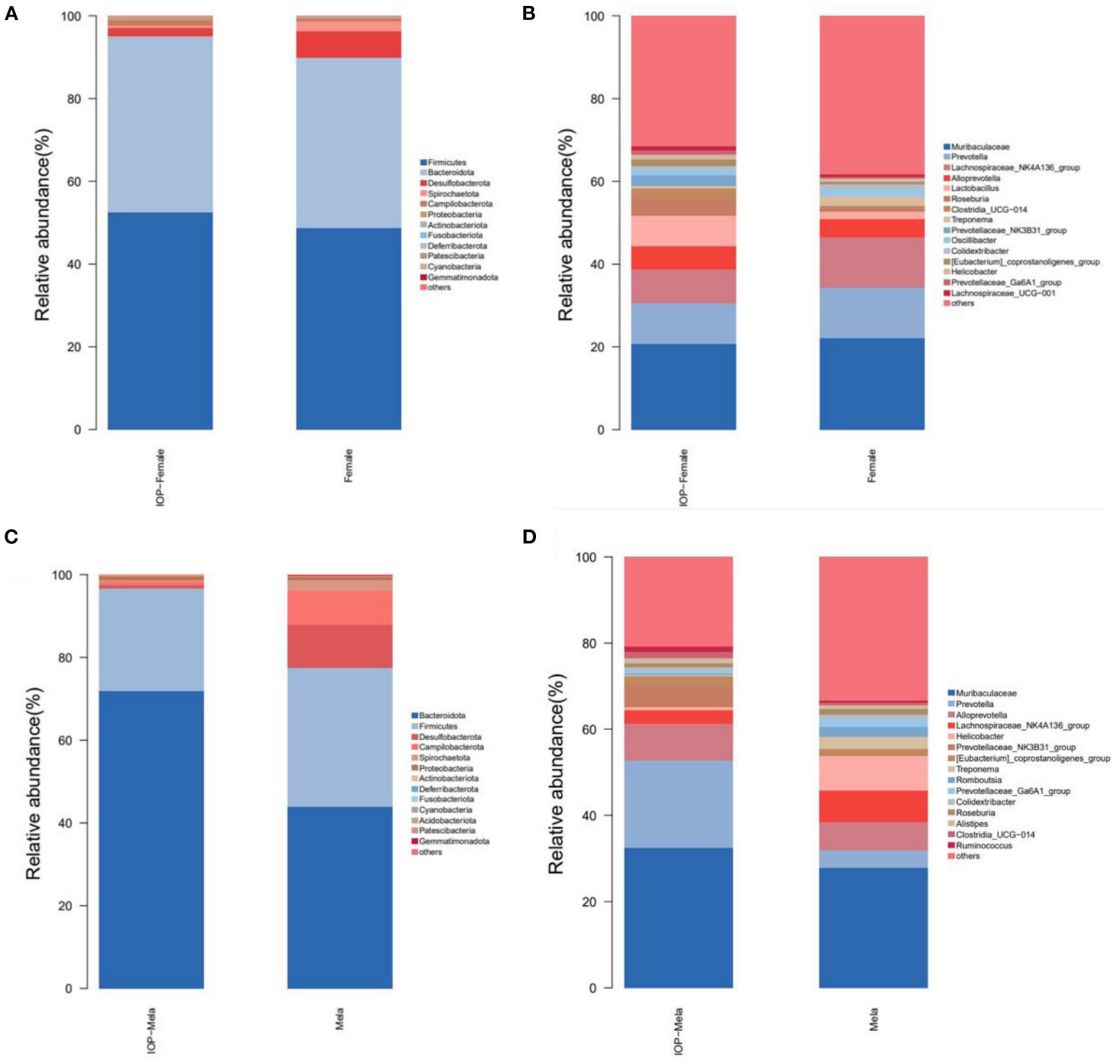
Histogram showing changes in the composition of intestinal microbes. **(A,B)** The first 15 different bacteria in female SD rats at phylum and genus levels. **(C,D)** The first 15 different microorganisms in male SD rats at phylum and genus levels.

The community structure of colorectal intestinal microbes of rats in the IOP group, as presented in the bar chart (Figure 5), showed a total of seven phyla with high abundance at phylum level. *Firmicutes* and *Bacteroidota* were the most dominant, accounting for 45.57 and 47.13% of the abundance respectively. There were, however, no significant differences in the F/B ratio for females, although that of males decreased. *Desulfobacterota* (3.28%), *Campilobacterota* (1.1%), *Spirochaetota* (1.6%), *Proteobacteria* (1.1%) and *Actinobacteriota* (0.2%) were some of the additional phyla that were also detected. At the genus level, the dominant genera mostly included *Muribaculaceae* (22.3%), *Prevotella* (12.2%), *Lachnospiraceae_NK4A136_group* (8.7%) and *Alloprevotella* (5.5%). In addition, the abundance of different species in each group was compared by Kruskal Wallis and it was found that, at the genus level, *Prevotellaceae_NK3B31_group, Lactobacillus* and *Roseburia* had significantly different abundances between groups.

A bar chart of the community structure of colorectal intestinal flora for SD rats within the control group is shown in Figure 5. At the phylum level, there were five phyla of relatively high abundance, and these included *Bacteroidota* (40.38%), *Firmicutes* (45.26%), *Desulfobacterota* (8.1%), *Campilobacterota* (2.9%) and *Spirochaetota* (2.0%). In particular, the abundance of *Firmicutes* in female rats was higher than for males, while that of *Campilobacterota* was lower compared with males. At the genus level, the five most abundant genera consisted of *Muribaculaceae* (22.3%), *Lachnospiraceae_NK4A136_group* (10.9%), *Prevotella* (8.25%), *Alloprevotella* (6.0%%) and *Helicobacter* (2.8%%). In this case, the beneficial bacteria *Prevotella* was higher in abundance in female rats than in male ones, while the harmful bacteria *Helicobacter* was more abundant in male rats. However, after IOP intervention, the diversity of female rats' microbiota increased, such as *Roseburia* and other beneficial bacteria. The F/B ratio and the proportion of Helicobacter and other harmful bacteria decreased in male rats.

### Differential analysis of intestinal microflora between male and female SD rats affected by IOP

**Figure 6:**
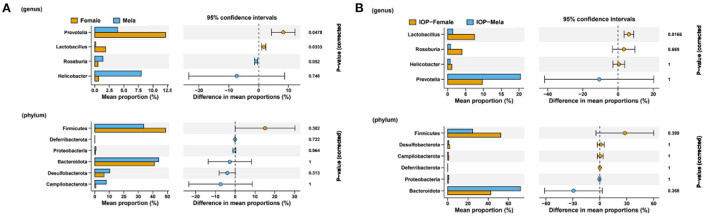
Comparison of intestinal microflora between male and female SD rats. Welch's two sample t-tests were performed to compare the control group **(A)** and the experimental group **(B)** with IOP intervention.

To evaluate whether differences in intestinal microbes after IOP treatment were influenced by the sex of the rats, Welch's two sample *t*-tests were used to explore differences between male and female SD rats at phylum level (Figure 6). The results, in this case, showed that, for the control group (without IOP treatment), *Firmicutes* (*P* = 0.3), *Lactobacillus* (*P* = 0.03) and *Roseburia* (*P* = 0.05) were higher in females than in males, while *Bacteroidota* (*P* = 1), *Campilobacterota* (*P* = 0.18) *Prevotella* (*P* = 0.04) and *[Eubacterium]siraeum_group* were lower when compared with males. Similarly, for the experimental group (treated with IOP), among the highly abundant species, *Firmicutes* and *Lactobacillus* were significantly higher in females than in males, while *Bacteroidota* (*P* = 0.36*)* was significantly lower compared with males. In contrast, among species of low abundance, *Desulfobacterota* (*P* = 1), *Deferribacterota* (*P* = 1) and *Roseburia* (*P* = 0.669) were significantly higher in females, while *Prevotella* (*P* = 1).

### Analysis of differences in dominant species between sexes

**Figure 7:**
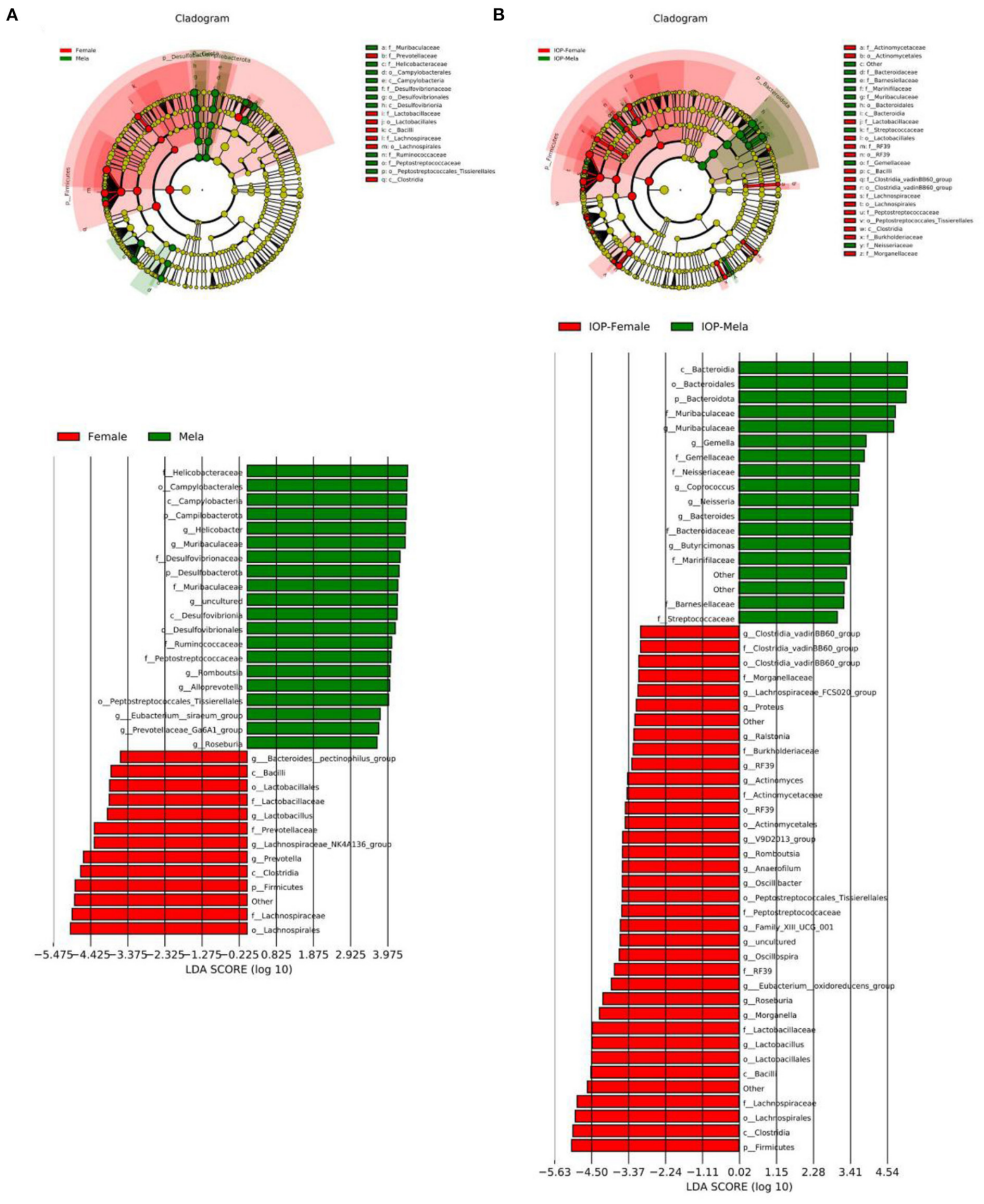
The control group **(A)** and the experimental group **(B)** with IOP interference were compared. Results of linear discriminant analysis were used to determine the dominant species after IOP interference.

Linear discriminant analysis Effect Size (LEFSE) was performed to evaluate the impact of sex-related differences on intestinal dominant microbes (Figure 7). It was found that, for the control, *Prevotella* and *Firmicutes* were the dominant intestinal microbes in female rats, while the dominant ones in males were *Helicobacte*r, *Muribaculaceae, Campilobacterota* and *Desulfobacterota*. In contrast, several genera of *Firmicutes* were dominant in female rats after IOP intervention, and, in particular, the proportion of *Clostridia, Lactobacillus* and *Roseburia* increased. As far as male rats were concerned, several *Bacteroidota* families and genera were dominant, with the proportion of *Muribaculaceae* families and genera particularly increasing. At the same time, the proportion of some pathogenic bacteria also decreased. Therefore, it was found that the changes in dominant bacteria occurring in females were smaller than those of males after IOP intervention. On the other hand, female intestinal microbes showed better diversity than that of males.

## Discussion

The diversity of gut microbes is associated with humans or animals health (29–31), and disruption of this relationship can have adverse effects (20, 32–34). For example, an imbalance in the normal gut flora may contribute to the development of type 2 diabetes mellitus, non-alcoholic fatty liver disease and other related metabolic diseases (35, 36). Similarly, sex hormones may induce changes in gut microbiota during ontogeny (37). In this case, too much androgen can cause dysregulation of the intestinal flora in females with polyovarian syndrome (38), while estrogen can mediate irritable bowel syndrome (IBS) by altering the permeability of the intestinal barrier (37). Interestingly, the polysaccharides from *Inonotus obliquus* exert certain biological activities, thereby allowing it to be used as a prebiotic to fight diseases such as diabetes, cancer and cardiovascular diseases (16). Previous research has already found that IOP could help alleviate acute endometritis (20) and improve the severity of both obesity (39) and chronic pancreatitis (40) by converting the diversity of intestinal flora to a healthier state and this, in turn, improves the antioxidant status, thereby reducing inflammation levels, and improving host metabolism (41).

The richness and diversity of intestinal microflora in male and female rats fed with IOP were analyzed by 16S rRNA sequencing. The results showed that there were differences in the regulation of intestinal microflora between male and female SD rats after *Inonotus obliquus* consumption, with these observations being consistent with a previous study which reported opposite changes in the correlation of α-diversity in the intestinal microflora of male and female SD rats (42). Similarly, as far as humans are concerned, sex-related differences such as total estradiol levels were shown to be negatively correlated with α diversity in healthy men, non-hyperandrogenic women as well as women with polycystic ovary syndrome (PCOS) (43).

Moreover, based on the weighted and unweighted UniFrac PCoA index, significant differences in both quantitative and qualitative indexes (weighted and unweighted UniFrac distance) were noted between male and female SD rats in the control group, while for the experimental group, differences between sexes were only visible for the qualitative index (unweighted UniFrac distance) (44).These findings could have been due to differences in the types of hormones secreted by female and male individuals, resulting in variations in the diversity of intestinal flora between mice of different sexes. Estrogen is an inhibitor of gastric acid production while testosterone is an inducer, estrogen may be more effective at repairing damage to the stomach wall as it reduces gastric blood flow in males (45). Estrogen has also been shown to influence the composition of intestinal microbes, with the reverse (i.e., the intestinal flora affecting the levels of estrogen) also being true (46).

Nevertheless, differences in the composition of intestinal microbes between male and female rats before and after being fed with IOP were compared and analyzed, with the results showing significant differences between male and female rats at phylum and genus level. The intestinal microbe for the IOP group was also significantly different compared with the control and at the same time, significant differences in the type of bacteria between sexes were also observed. *Roseburia* was significantly more abundant in females than in males. This genus is able to utilize fructan and increase glucagon-like peptide-1, thereby reducing the risk of obesity (43), with this function being important as obesity has been reported to promote high androgen production, which could lead to the development of polycystic ovarian syndrome (47). Based on the results, it was further speculated that *Roseburia* could reduce the secretion of sex hormones. In male rats that were given IOP, *Muribaculaceae* (48), which can regulate testosterone induced benign prostatic hyperplasia (43, 49). However, *Prevotella*, which was positively correlated with testosterone, was higher in abundance than in females while the F/B ratio was lower compared with that of females. Previous studies have also shown that the F/B ratio is related to intestinal environment stability and body health status (50). In this context, the results of this study showed that IOP could reduce the ratio of F/B in the intestinal flora of male and female rats, and this can promote the stability of the intestinal environment and lower the Body Mass Index. Therefore, altogether, the findings suggest that changes in intestinal microbes could be dependent on sex and sex hormones, as reported by previous research results (51).

The relative abundance of harmful bacteria like *Desulfobacterota* and *Campilobacterota* decreased after using IOP. The level of *Desulfobacterota* was reported to be positively correlated with body weight and lipid level (52). The current results showed that IOP reduced the relative abundance of *Desulfobacterota* in both male and female rats and therefore, it was hypothesized that IOP could intervene with the body health by regulating intestinal microbes. It has been reported that the abundance of *Campilobacterota* was significantly increased in the NLRP6 inflammatory body regulating chronic alcoholic liver disease in mouse models (53). In the results of this study, IOP reduced the abundance of harmful bacteria such as *Campilobacterota* in male rats, and hence, it was shown that IOP could reduce inflammation by improving intestinal flora. Additionally, changes in intestinal microbe between male and female rats may also affect the digestion of polysaccharide compounds (54), vitamin synthesis (55), and the expulsion of pathogenic microorganisms (56) etc. Hence, these results may further explain differences in the incidence of intestinal metabolic syndrome (44), and obesity (57) between sexes as they are all associated with changes in gut flora.

Differences in dominant bacterial species in the intestinal flora of male and female rats were also analyzed after IOP consumption. It was found that *Lactobacillus, Roseburia* and *Clostridia* were dominant in females of the IOP group as previously reported (19, 57). For instance, in the case of females from the experimental group, *Lactobacillus* mainly consumed polysaccharides, and lactate, the end product of its fermentation, could be used as an energy source by *Roseburia* to produce butyrate. Additionally, bacterial fermentation of polysaccharides leads to the production of acidic products such as lactic acid and short chain fatty acid (SCFAs) which reduce the pH of the colorectum to influence the microbial community composition of the gastrointestinal tract. In this context, it has been reported that higher acidic conditions are conducive to the growth of Firmicutes such as *Roseburia*, which produce butyric acid, thereby reducing the proliferation of acid-sensitive Bacteroidia species (58). The presence of *Lactobacillus* can not only reduce intestinal permeability and increase the release of anti-inflammatory factors in steroid deficient mice (59), but also prevent the adhesion of *Neisseria gonorrhoeae*, thus protecting the host from infections (60). In addition, *Clostridia* not only participates in bile acid (BA) biotransformation (61), but also converts glucocorticoids to androgens (62). In studies of the potential role of gender-dependent autoimmunity in gut microbiota, it was suggested that hormones could interact with gut microbiota to mediate sexual dimorphism (63). In male rats, polysaccharide-degrading bacteria were mainly dominated by *Bacteroidia*, such as *Muribaculaceae* (64), which could increase the expression of AhRR in intestinal immune cells as well as *Bacteroides* which could synthesize conjugated linoleic acid (CLA). The latter can regulate immune functions, lower blood lipids and exert anti-diabetic as well as anti-inflammatory effects (65, 66).Through comparative analysis examining how dominant bacteria varied between sexes after consuming IOP, it was found that IOP could improve the activity of dominant bacteria in intestinal microbes, and these results could form the basis of a future approach whereby bacterial species are selected based on sex-related differences to reshape the intestinal microenvironment, while providing ideas for metabolic analysis.

## Conclusion

The results of this study suggest that there are indeed sex-related differences in the diversity of intestinal microbes after IOP interference and that these differences could be associated with the incidence of metabolic diseases. IOP could not only produce different effects on the intestinal microbiota based on the sex of the animals, but also improve the activity of dominant microbes. The current findings could also provide a basis for designing new interventions aimed at preventing metabolic diseases by regulating differences in the intestinal microbes of different sexes through IOP, improving the microenvironment of intestinal microbes and enhancing the vitality of dominant species.

## Data availability statement

The datasets presented in this study can be found in online repositories. The names of the repository/repositories and accession number(s) can be found below: Sequence Read Archive of the National Center for Biotechnology Information repository; PRJNA862723.

## Ethics statement

The animal study was reviewed and approved by Animal Care Office of Chengdu Normal University.

## Author contributions

Conceptualization: BH, XH, and JT. Methodology: BH, WZ, and XD. Software: XL. Validation: BH and GW. Formal analysis: XH, JT, and ML. Investigation: YG, MS, RS, and XD. Resources, supervision, and funding acquisition: BH and SL. Data curation: BH, XH, JT, and SL. Writing—original draft preparation and visualization: BH and YM. Writing—review and editing: YM, BH, and SL. Project administration: GW. All authors have read and agreed to the published version of the manuscript.

## Funding

This work was supported by the Chengdu Normal University scientific research project [No.111-153701] (Acceptor: BH), Public welfare project of pet chaperoning [No.107-261277] (Acceptor: BH), and Chengdu Normal University agricultural ecology and green food development project [CSCXTD2020B11] (Acceptor: SL).

## Conflict of interest

The authors declare that the research was conducted in the absence of any commercial or financial relationships that could be construed as a potential conflict of interest.

## Publisher's note

All claims expressed in this article are solely those of the authors and do not necessarily represent those of their affiliated organizations, or those of the publisher, the editors and the reviewers. Any product that may be evaluated in this article, or claim that may be made by its manufacturer, is not guaranteed or endorsed by the publisher.
